# Detrimental ELAVL-1/HuR-dependent GSK3β mRNA stabilization impairs resolution in acute respiratory distress syndrome

**DOI:** 10.1371/journal.pone.0172116

**Published:** 2017-02-14

**Authors:** Olivia Hoffman, Nana Burns, István Vadász, Holger K. Eltzschig, Michael G. Edwards, Christine U. Vohwinkel

**Affiliations:** 1 Department of Pediatrics, University of Colorado at Denver, Aurora, Colorado, United States of America; 2 Developmental Lung Biology, Cardio Vascular Pulmonary Research Laboratories, Division of Pulmonary Sciences and Critical Care Medicine, Division of Pediatric Critical Care, Departments of Medicine and Pediatrics, University of Colorado, Anschutz Medical Campus, Aurora, Colorado, United States of America; 3 Department of Internal Medicine, Justus Liebig University, Universities of Giessen and Marburg Lung Center, Member of the German Center for Lung Research, Giessen, Germany; 4 Organ Protection Program, Department of Anesthesiology, University of Colorado, School of Medicine, Aurora, Colorado, United States of America; University of Alabama at Birmingham, UNITED STATES

## Abstract

A hallmark of acute respiratory distress syndrome (ARDS) is accumulation of protein-rich edema in the distal airspaces and its removal is critical for patient survival. Previous studies have shown a detrimental role of Glycogen Synthase Kinase (GSK) 3β during ARDS via inhibition of alveolar epithelial protein transport. We hypothesized that post-transcriptional regulation of GSK3β could play a functional role in ARDS resolution. To address this hypothesis, we performed an *in silico* analysis to identify regulatory genes whose expression correlation to GSK3β messenger RNA utilizing two lung cancer cell line array datasets. Among potential regulatory partners of GSK3β, these studies identified the RNA-binding protein ELAVL-1/HuR (Embryonic Lethal, Abnormal Vision, Drosophila-Like) as a central component in a likely GSK3β signaling network. ELAVL-1/HuR is a RNA-binding protein that selectively binds to AU-rich elements of mRNA and enhances its stability thereby increasing target gene expression. Subsequent studies with siRNA suppression of ELAVL-1/HuR demonstrated deceased GSK3β mRNA and protein expression and improved clearance of FITC-albumin in A549 cells. Conversely, stabilization of ELAVL-1/HuR with the proteasome inhibitor MG-132 resulted in induction of GSK3β at mRNA and protein level and attenuated FITC-albumin clearance. Utilizing ventilator-induced lung injury or intra-tracheal installation of hydrochloric acid to induce ARDS in mice, we observed increased mRNA and protein expression of ELAVL-1/HuR and GSK3β. Together, our findings indicate a previously unknown interaction between GSK3β and ELAV-1 during ARDS, and suggest the inhibition of the ELAV-1- GSK3β pathways as a novel ARDS treatment approach.

## Introduction

Acute lung injury (ALI) is an acute form of lung injury that manifests itself in patients as acute respiratory distress syndrome (ARDS) that is characterized by an acute onset of mild to severe hypoxemia, pulmonary edema not explained entirely by fluid overload or cardiac disease and bilateral chest X-ray opacities[1]. As an estimate of 200,000 patients are diagnosed with ARDS annually in the United States, ARDS carries a significant disease burden both in regards to morbidity and mortality [2], especially as there is growing evidence that severe ARDS has long-term physical and psychological sequela [3]. Current therapies for ARDS are mostly supportive but do not target the primary pathophysiologic mechanism *per se*. One of the major features of ARDS is the accumulation of protein-rich alveolar edema [4] leading to severely impaired gas exchange and thus to alveolar hypoxia and systemic hypoxemia. In patients, that died from ARDS, protein concentration in their edema fluid was triple compared to survivors of the disease [5]. Therefore, the capability to clear proteinaceous exudate from the alveolar space is critical for the resolution of ARDS [6]. Protein clearance from the distal air spaces is facilitated by an active endocytotic process mediated by megalin a 600 kDa glycoprotein member of the low-density lipoprotein (LDL)-receptor superfamily [7]. Megalin in turn is negatively regulated by glycogen synthase kinase 3β (GSK3β) and our data indicate that the transcription of GSK3β is induced in several murine models of ALI. GSK3, a serine/threonine protein kinase signaling molecule, is widely expressed in various cell types. The 2 isoforms in mammals GSK3α (51 kDa) and GSK3β (47 kDa) are 98% structurally identical [8, 9]. Little is known about the functional differences between the two [10] but GSK3β is the more abundant and more widely studied of the two, as several studies have linked GSK3β hyperactivation to various pathological conditions, including diabetes mellitus, inflammation, pulmonary hypertension and Alzheimer’s disease [9, 11–13]. However, little is known about the transcriptional regulation of GSK3β. In the present study, utilizing an *in silico* approach to discover potential regulators of GSK3β, we identified Embryonic Lethal, Abnormal Vision, Drosophila-Like 1 / Human antigen R (ELAVL-1/HuR) as a potential upstream regulator of GSK3β. ELAVL-1/HuR is part of a family of RNA-binding proteins. RNA-binding proteins are key regulators of gene expression and ELAVL-1/HuR selectively binds AU-rich elements (AREs) found in the 3' untranslated regions of mRNAs [14]. By binding to ARE regions ELAVL-1/HuR plays a role in mRNA stabilization[15].We subsequently validated the GSK3β- ELAVL-1/HuR interaction in vitro and several murine models of ALI. Mortality in ARDS can at least partially be attributed to failure to clear the protein rich edema from alveolar space. Therefore understanding the regulatory mechanism that govern protein transport across alveolar epithelial cells and the alveolo-capillary barrier is critical for the development of new therapies for ARDS [6].

## Materials and methods

### Materials

Unless otherwise noted, chemicals were obtained from Sigma (St. Louis/ MO, USA), MG-312 was purchased from Calbiochem San Diego/CA USA), ELAVL-1/HuR antibody and ELAVL-1/HuR siRNA were purchased from Santa Cruz Biotechnology (Dallas/TX, USA), GSK3β antibody form Cell signalling (Danvers, MA, USA) and Lipofectamin RNAimax were from Life Technologies (Carlsbad/CA, USA). ECL Detection Kit and nuclear and cytoplasmatic Extraction kit were obtained from Thermo Fisher Scientific (Grand Island/ NY, USA). RNA extraction kit was from Qiagen (Valencia/CA, USA).

### In silico analysis of GSK3β and ELAVL-1/HuR

Correlation analysis was previously described in [16]. *Pearson correlation studies-*Lung cancer cell line gene expression data sets from the Cancer Cell Line Encyclopedia (CCLE) (GEO#GSE36133, 166 unique cell lines) and UT Southwestern Medical Center (GEO#GSE4824, 76 unique cell lines) were downloaded from Gene Expression Omnibus (GEO) and used to find genes correlated to GSK3βexpression. There are 42 cell lines shared between the lung cancer cell line datasets (~60% of the UT study) and both use the same platform (Affymetrix) to assess global gene expression. An absolute r-value of greater than 0.55 (Pearson) was used to define significant correlation to GSK3β independently in each dataset. There were 377 and 355 transcripts correlated to GSK3β (|r|>0.55, Pearson) in the CCLE and UT datasets respectively, with 120 of these being common to both datasets. We have uploaded an excel spreadsheet as a supplementary table (S1 Table) containing the Affy probeset ID and additional annotation for each commonly correlated transcript along with their associated r- and p- values for each dataset.

*Bioinformatics analysis*-The 120 transcripts correlated to GSK3β were analyzed for over-representation of biological functions, pathways and networks using the bioinformatics software Ingenuity Pathways Analysis (IPA) (www.ingenuity.com). IPA takes a select list of genes and creates limited, interconnected networks (35 genes maximum) based on evidence of direct or indirect biological relationships contained in the Ingenuity Knowledge Base. The IPA network algorithm seeks to maximize the interconnectivity within a group of selected genes and scores networks based on a right tailed Fisher’s exact test that calculates the probability that the given relationships can be explained by a random model. The networks do not include all possible relationships for each member, because of size constraints placed on the network, and specific genes may appear in multiple networks.

### Cells line and primary cells

A549 cells (ATCC, CCL185) were cultured in DMEM with 4.5 g/L glucose and stable L-glutamine, 10% fetal bovine serum (FBS) and 1% penicillin/streptomycin mix (all from Corning Cellgro, Manassas/VA, USA). Cells were incubated in a humidified atmosphere of 5% CO2/95% air at 37°C.

### Real time RT qPCR

RNA was isolated from A549 cells using a Qiagen RNeasy kit (Qiagen, Valencia/CA, USA) according to the manufacturer’s instructions. cDNA was obtained using iScript cDNA Synthesis Kit (Bio-Rad, Hercules/CA, USA). qPCR was performed with iTaq SYBRGreen Supermix (Bio-Rad) using an iCycler (Bio-Rad). Following primer sequences were used: GSK3β (hs) sense AAGGATTCGTCAGGAACAGGA, GSK3β (hs) antisense CTGCTTGAATCCGAGCATGA, GSK3β (mm) sense TTGGCCACTGTCGCTATTGT, GSK3β (mm) antisense TCCTTCCTTTGTCACTCGGC, ELAVL-1/HuR (hs) sense TTGCAAGCTTGTGGAAGGAT, ELAVL-1/HuR (hs) antisense TTACACACGGGTCAAAAGGG, ELAVL-1/HuR (mm) sense GTTCTTCCGCCTACTTCTGG, ELAVL-1/HuR (mm) antisense AAAGCTGGCCACATAAACCA.

### RNA interference of ELAVL-1/HuR

A549 cells were cultured until 40–60% confluence. Human siRNA (sc-35619, Santa Cruz Biotechnology, Dallas/TX, USA) was transfected using Lipofectamine RNAiMAX (Life technologies Invitrogen) according to the manufacturer. Scrambled sequence siRNA (Santa Cruz Biotechnology) and cy3 labelled negative control (Life technologies Darmstadt, Germany) were used as controls. Cells were used 48 hours after transfection.

### Binding, uptake of FITC- labelled albumin

Method has been previously described in detail [7]. A549 cells grown on 6 well plates were used. After removal of the culture medium, dish was washed and pre-incubated with Dulbecco’s phosphate buffered saline containing 5 mM glucose, 0.1 mM CaCl_2_ dihydrate and 0.5 mM MgCl_2_ 6H_2_O (DPBS-G) for 15 min at experimental conditions. Then DPBS-G buffer containing 50 μg/ml FITC-albumin was added to each dish and cells were incubated at 37°C or 4°C for 1 hour. At the end of the experiments cells were rinsed three times with ice cold PBS and incubated with 0.5 ml of ice-cold Solution X (DPBS-G, 0.5 mg/ml trypsin, 0.5 mg/ml proteinase K, 0.5 mM EDTA before being scraped. After centrifugation at 4° C for 5 min samples were taken from the supernatant to asses the bound fraction. After the supernatant was aspirated, the pellet was solubilised in 0.5 ml 0.1% Triton-X-100 (in PBS buffer without CaCl_2_ and MgCl_2_) for 30 min at room temperature and centrifuged for 5 min at 8000 rpm. Fluorescence was detected using a BioTek Synergy 2 fluorescence spectrophotometer (BioTek, Winooski/VT, USA) at an excitation wavelength of 500 nm and an emission wavelength of 520nm.

### Cell fractionation

The experiment was terminated by placing the cells on ice and washing them twice with ice-cold PBS. Nuclear and cytoplasmatic extraction kit (Thermo Scientific Fisher) was used according to the manufacturer’s instructions. Cells were scraped in homogenization buffer with 1 mM phenylmethanesulfonyl fluoride.

### Western blot analysis

Protein concentration was quantified by Bradford assay (Bio-Rad, Hercules/CA USA), and proteins were resolved in 12% polyacrylamide gel. Thereafter, proteins were transferred to nitrocellulose membranes (Santa Cruz Biotechnology Dallas/TX, USA) using a semi-dry transfer apparatus (Bio-Rad, Munich, Germany). Incubation with ELAVL-1/HuR (HuR 3A2) antibody 1:5000 (sc-5261, Santa Cruz Biotechnology) respectively GSK3β antibody 1: 2000 (cell signaling #9315) were performed overnight at 4°C. Blots were developed with a chemiluminescence detection kit (Perkin Elmer Inc. Waltham/MA USA), as recommended by the manufacturer.

### Animal models of acute lung injury

Animal experiments were approved by the local institutional animal care and use committee (University of Colorado, Anschutz Medical Campus), protocol number B104914(06)1D. All experiments were conducted in wild type C57/B6 mice age 8–10 weeks, weight 20–25 grams.

#### Ventilator Induced Lung Injury (VILI)

Ventilator induced lung injury was described previously [17, 18]. Briefly, ALI was induced with mechanical ventilation utilizing high inspiratory pressure levels (45 mbar) during pressure-controlled ventilation. Animals were anesthetized with pentobarbital (70 mg/kg i.p. for induction; 20 mg/kg/h for maintenance) and placed on a temperature-controlled heated table with a rectal thermometer probe attached to a thermal feedback controller to maintain body temperature at 37°C. Tracheotomy and mechanical ventilation was performed as described previously[19]. In short, the tracheal tube was connected to a mechanical ventilator (Siemens Servo 900C, with pediatric tubing). Mice were ventilated in a pressure-controlled ventilation mode at the inspiratory pressure levels (45 mbar for VILI group 15 mbar respectively for control group) over 4 hours. Respiratory rate and inspiratory/expiratory time ratios and PEEP were kept constant in both groups. All animals were ventilated with 100% inspired oxygen and PEEP of 3.

#### Acid aspiration induced lung injury

Acid aspiration model of ALI was described previously [20]. Briefly, animals were anesthetized with isoflurane (1–3% for maintenance; up to 5% for induction) and suspended by their incisors from a custom made 45° angled mount. A 22 G catheter was guided 1 cm below the vocal cords via guide wire using a small animal laryngoscope (Penncentury, Wyndmoor/PA, USA) and 50μl of 0.125 M hydrochloric acid (HCl) were instilled. Control animals received 50μl of 0.9 M NaCl.

### Data analysis

Data were compiled from at least three independent, replicate experiments, each performed on separate cultures or animals. The mRNA responses are displayed as “fold-changes.” Data are expressed as means ± SEM. When comparisons were performed between two groups, significance was evaluated by Student's t test, and when more than two groups were compared analysis of variance was used, followed by Dunnett's test. p < 0.05 was considered significant.

## Results

### ELAVL-1/HuR is a novel upstream regulator of GSK3β

**Fig 1:**
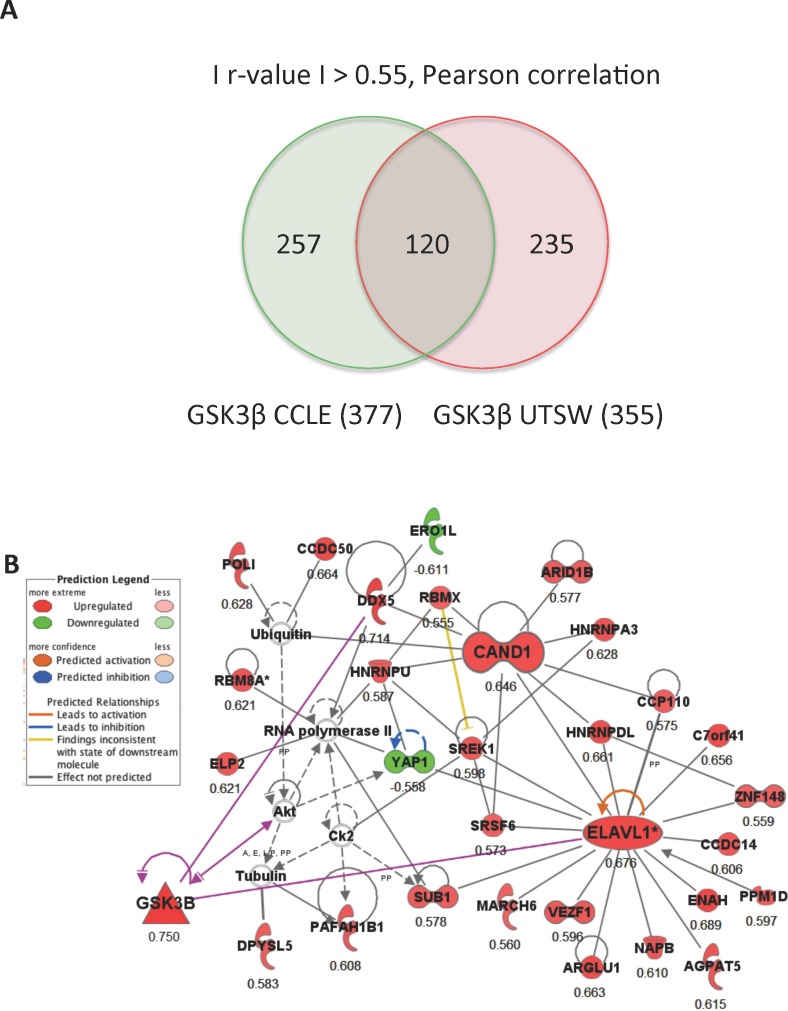
*In silico* identification of ELAVL-1/HUR as a potential regulator of GSK3β. (A) Gene expression data sets from the Cancer Cell Line Encyclopedia (CCLE) and UT Southwestern Medical Center (UTSW) were analyzed independently to find genes correlated to GSK3β expression. We identified 120 common transcripts with correlation coefficient |r|> 0.55 in both lung cell data sets (B) Gene network analysis based on previously known direct (solid lines) and indirect (dashed lines) biological connections for the identified transcripts with a positive (red) and negative correlation (green) r-value >0.55) to GSK3β expression in two, independent lung cancer cell line datasets.

**Fig 2:**
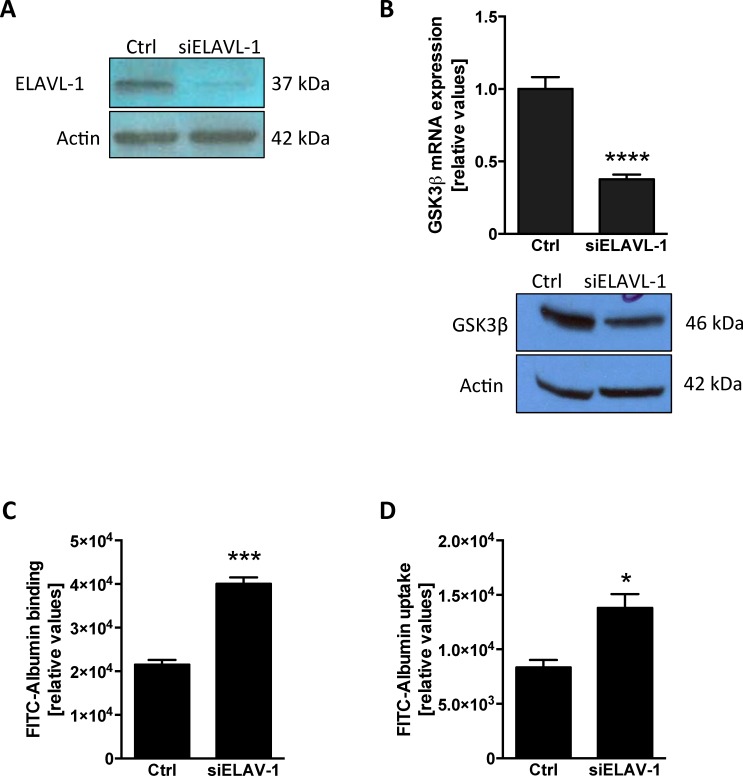
Suppression of ELAVL-1/HUR leads to down regulation of GSK3β *in vitro* and subsequently increased of epithelial albumin clearance. **(**A) ELAVL-1/HUR protein expression and suppression in nuclear cell lysate in cells treated with ELAVL-1/HUR siRNA. (B) mRNA and protein expression of GSK3β in cells transfected with siELLAVL-1. Binding (C) and uptake (D) of FITC labelled albumin All experiments were conducted in A549 cells, n 3–4; data represent the mean ± SEM * p< 0.05, ***p<0.001, ****p<0.0001.

**Fig 3:**
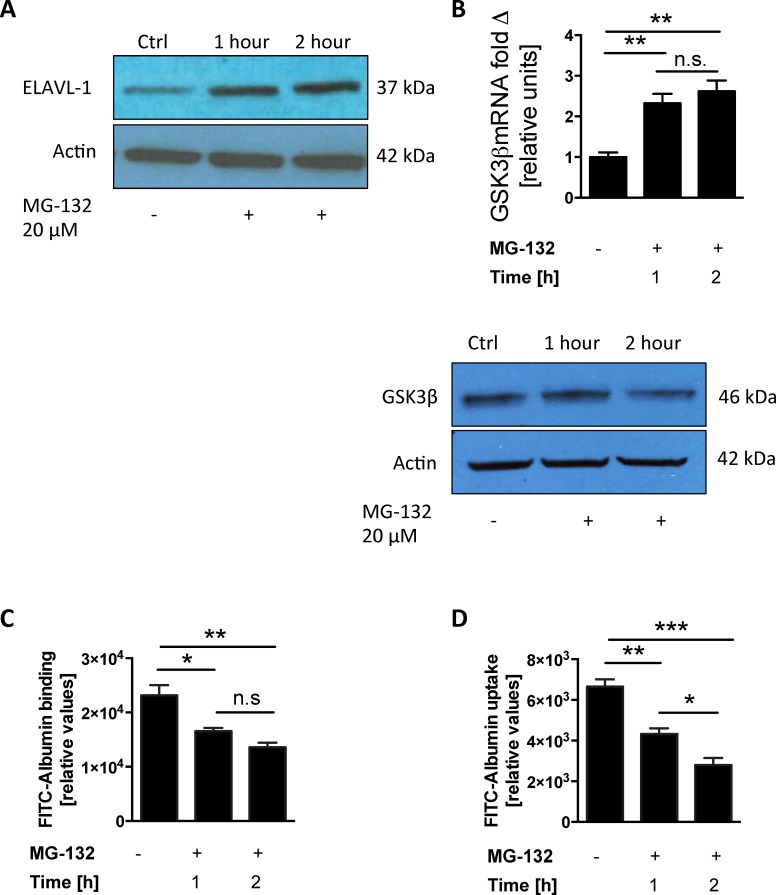
Stabilization of ELAVL-1/HUR up regulates GSK3β in vitro and attenuates epithelial albumin clearance. Cells were incubated with 20 μM of the lysosomal inhibitor MG-132 at indicated time points to stabilize ELAV-1. (A) ELAVL-1/HUR protein expression was determined in the nuclear cell lysate via western blot. (B) mRNA and protein expression of GSK3β in cells treated with MG-132. Binding (C) and uptake (D) of FITC labelled albumin All experiments were conducted in A549 cells, n = 4; data represent the mean ± SEM * p< 0.05, **p<0.01, ***p<0.001, n.s. = non significant.

To investigate the regulatory mechanism of GSK3β expression we performed comprehensive pathway analysis and built a gene interaction network based on publicly available gene expression datasets containing an extensive number of epithelial lung tumor cell lines. We identified 120 common transcripts, out of 612 transcripts independently correlated to GSK3β (r >0.55, Pearson), in two different datasets (Fig 1A). There was complete concordance in the direction of correlation between the two datasets for the 120 common genes (S1 Table). When we used IPA to connect these genes based on previously known biological associations, we found that the top network is enriched in associations within the list of correlated genes (p = 1 × 10^−6970^, Fisher's exact test), with the ELAVL-1/HuR central to these connections (Fig 1B). We hypothesized that ELAVL-1/HuR mediates alveolar epithelial protein transport through the regulation of GSK3β expression, so we first examined this relationship *in vitro*, by evaluating if genetically inhibiting ELAVL-1/HuR would affect the expression of GSK3β. We transfected A549 cells with ELAVL-1/HuR siRNA (Fig 2A) and found that suppression of ELAVL-1/HuR significantly decreased GSK3β mRNA levels compared to control from 1 ± 0.08 to 0.377 ± 0.03 (n = 3) (Fig 2B). This corresponded with decreased GSK3β protein expression in cells transfected with siHUR (Fig 2B). In a next step, we examined the impact of ELAVL-1/HuR suppression and subsequent decrease in GSK3β mRNA levels on alveolar epithelial protein clearance by measuring albumin binding and uptake (relative units). We found that suppression of ELAVL-1/HuR with siRNA interference significantly augmented binding 21535 ± 1075 in controls versus 40074 ± 1432 in siELAVL-1/HuR transfected cells (n = 4) (Fig 2C) and uptake 8342 ± 687 in controls versus 13797 ± 1253 in siELAVL-1/HuR transfected cells (n = 4) (Fig 2D) of FITC-labeled albumin. The proteosomal inhibitor MG-132 has been shown to enhance ELAVL-1/HuR expression at mRNA and protein level [21]. When ELAVL-1/HuR was stabilized by MG-132 (Fig 3A) we found increased GSK3β mRNA levels after 1 hour (2.32±0.23) and 2 hours (2.62±0.27) (n = 4) compared to control (Fig 3B), correspondingly MG-132 increased GSK3β protein expression (Fig 3B). Incubation with MG-132 subsequently reduced epithelial binding (Fig 3C) with 23189± 1852 (controls) versus 16577 ± 592 (after 1 hour) and 13584± 861 (2 hours) respectively (n = 4) and uptake with 6655 ± 352 (control) versus 4326 ± 282 (1hour) and 2795±354 (2hours) (n = 4) (Fig 3D) of FITC-labeled albumin in a time dependent manner.

### ELAVL-1/HuR and GSK3β in murine models of acute lung injury

**Fig 4:**
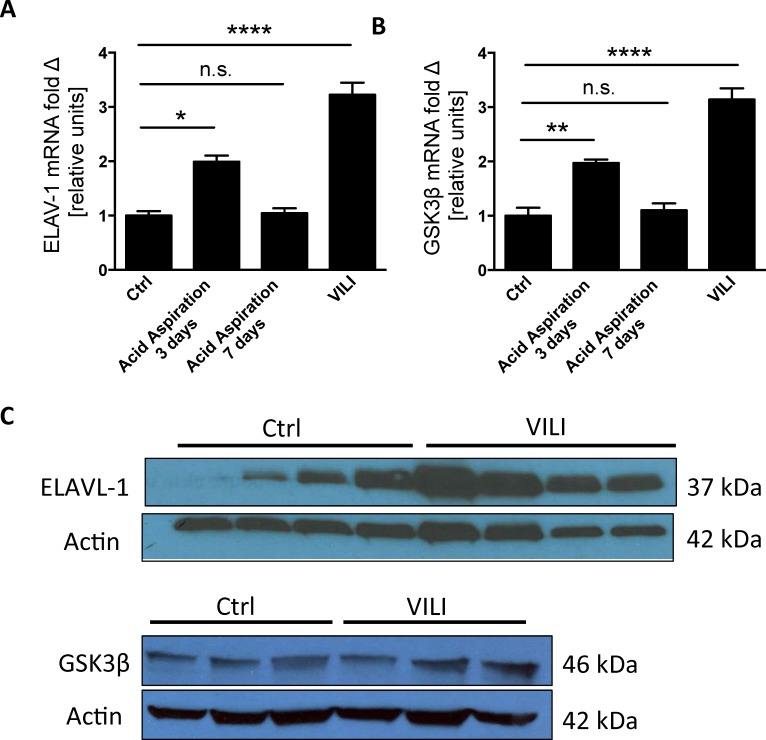
ELAVL-1/HUR and GSK3β expression in murine models of acute lung injury. ELAVL-1/HUR (A) and GSK3β (B) mRNA expression was determined in control animals and animals subjected to acute lung injury through acid aspiration of VILI. (C) ELAVL-1/HUR protein expression in nuclear cell lysate in control animals and animals subjected to VILI. All experiments were conducted in in primary alveolar epithelial cells., n = 4; data represent the mean ± SEM * p< 0.05, **p<0.01, ****p<0.0001, n.s. = non significant.

**Fig 5:**
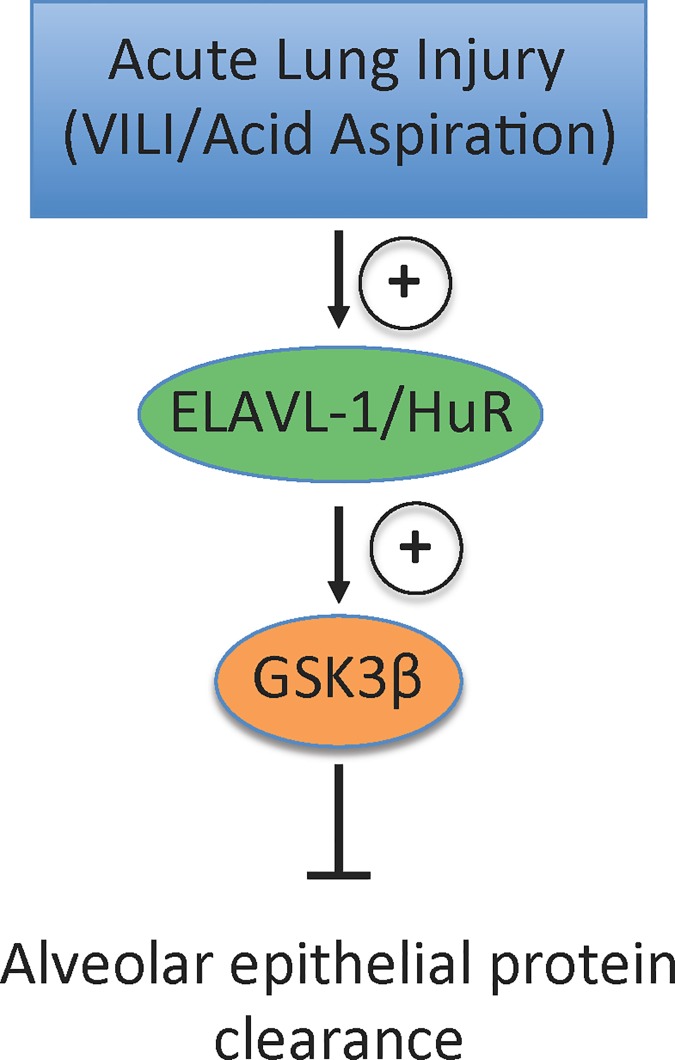
Summary- acute lung injury induces ELAVL-1/HuR transcription, which augments GSK3β mRNA mediated impairment of alveolar protein clearance.

After demonstrating that ELAVL-1/HuR regulates GSK3β *in vitro* we set out to validate your findings *in vivo*. As exudation of protein rich edema in the intra-alveolar space is a hallmark of ALI we utilized two different model of murine ALI: 1) VILI to examine the initial exudate phase of ALI and 2) acid aspiration to capture the resolution and repair phase of ALI. In the acid aspiration group ELAVL-1/HuR (1.99 ± 0.11) (Fig 4A) and GSK3β (1.97 ± 0.06) (Fig 4B) mRNA were significantly increased when compared to control animals (1 ± 0.08 for ELAVL-1/HuR and 1± 0.15 for GSK3β) at 3-days after acid aspiration. At the 7-day time point both ELAVL-1/HuR (Fig 4A) and GSK3β (Fig 4B) mRNA levels had returned to baseline (1.04 ± 0.09 for ELAVL-1/HuR and 1.1 ± 0.15 for GSK3β). In the VILI group we also found increased ELAVL-1/HuR with 3.22 ± 0.22 (Fig 4A) and GSK3β with 3.14 ± 0.42 (Fig 4B) mRNA levels compared to control (n of 4 animals per group). Consistently with the HuR and GSK3β mRNA increase, HuR respectively GSK3β protein expression were also elevated in the lung homogenates of the VILI group compared to the control ventilated animals (Fig 4C). Interestingly the increase of ELAVL-1/HuR (Fig 4A) and GSK3β (Fig 4B) mRNA in the animals subjected to VILI exceeded those in the acid aspiration group suggesting that the ELAVL-1/HuR–GSK3β axis seems to affect preferentially the initial phase of ALI. Collectively our findings may suggest ELAVL-1/HuR is induced in the lung epithelium during ALI and augments GSK3β mRNA levels, which in turn impairs epithelial protein clearance (Fig 5).

## Conclusions

Clearing the intralveolar space of proteinaceous edema is of critical importance in pathogenesis of ARDS and tied to patient survival [5]. The protein rich exudate found in alveolar fluids from patients with ARDS propagates persistence of pulmonary edema and inflammation and thus impairs resolution of lung injury. Despite the importance of alveolar protein clearance, the regulation of alveolar epithelial protein transport remains obscure. Therefore, identifying the mechanisms that govern the impaired resolution of proteinaceous exudate in ARDS is of high clinical importance.

We previously found that megalin, a member of the low-density lipoprotein (LDL)-receptor superfamily, regulates the binding and uptake of albumin the most abundant plasma protein from the intra-alveolar space [7]. The serine/threonine kinase GSK3β negatively regulates megalin and thus inhibits epithelial protein transport. GSK3β phosphorylates a PPPSP motif at the cytoplasmatic tail of megalin leading to its subsequent downregulation [22]. GSK3β dysregulation, particularly hyperactivation, has been associated with various pathological conditions, including diabetes mellitus, inflammation, pulmonary hypertension and Alzheimer’s disease [9, 11–13]. GSK3β has furthermore been implicated in hyperoxia induced lung injury [23] and LPS induced ALI [24]. These studies focus on the downstream effect of GSK3β on NF-kappa B and wnt signaling in neutrophils and macrophages rather than on the epithelium. However, GSK3β has been described to play a role in pulmonary endothelial permeability via reactive oxygen/nitrogen species [25].

Through *in silico* analysis we found that ELAVL-1/HuR expression is tied to GSK3β expression therefore identifying ELAVL-1/HuR as a potential regulator of GSK3β. ELAVL-1/HuR has been implicated macrophage function in ALI [26] however little is known about the function of ELAVL-1/HuR in the alveolar epithelium in ALI. We show for the first time that ELAVL-1/HuR regulates epithelial protein clearance. Our *in vivo* studies are restricted in that our analysis of ELAVL-1/HuR and GSK3β mRNA and protein expression expression were performed in whole lung tissue. The use of primary alveolar epithelial cells would be clearly superior. An additional limitation is the lack of an ELAVL-1/HuR overexpression construct, which why we reverted to the proteosomal inhibitor MG-132 to achieve ELAVL-1/HuR stabilization. Although MG-132 has been previously described to stabilize ELAVL-1/HuR [21], we are aware that MG-132 is rather unspecific. As a proteosomal inhibitor MG-132 likely has unrelated effects on autophagy, which has been reported to play a role in several mechanisms of ALI such as mitochondrial reactive oxygen species generation or epithelial cell death [27–29], which we did not observe in our *in* vitro system (S1 Fig)

ELAVL-1/HuR is a RNA binding protein and has been shown to modify mRNA post-translationally by increasing mRNA stability. ELAVL-1/HuR has been shown to modify tumor growth in non-small cell lung carcinoma and cigarette smoke induced associated airway inflammation [30–32]. In endothelial cell lines ELAVL-1/HuR induces intercellular adhesion molecule-1 (ICAM-1) and interleukin-8 (IL-8) after TNF α stimulation [33]. Our findings add to the growing body of evidence that post-transcriptional modification through RNA binding proteins may play are significant role in the pathophysiology of ALI [26, 34, 35]. Interestingly, CAND-1 (Cullin-Associated And Neddylation-Dissociated 1) the gene with the biggest correlation coefficient in our screen after the top-hit ELAVL-1/HuR also affects post-transcriptional regulation by inhibiting ubiquitination and therefore degradation by preventing the formation of E3 complex [36]; however, CAND-1 is only slightly expressed in lung tissue, which is why we focused on ELAVL-1/HuR [37].

In summary, to the best of our knowledge we describe for the first time a interaction between ELAVL-1/HuR and GSK3β and their role in alveolar epithelial protein clearance. This interaction was initially identified through in silico analysis, which we then validated in vitro and in murine models of ALI. Inhibiting the ELAVL-1/HuR -GSK3β axis, especially at the level of GSK3β as there several GSK3β inhibitors already in clinical practice [38], could be an intriguing, novel approach to ARDS therapy as it directly targets one of the major underlying pathomechanisms of the disease. Testing already clinically established GSK3β inhibitors in murine models of ALI would be a first step in this direction.

## Supporting information

S1 Table In silico analysis of potential GSK3β interactions. (PDF)

S1 Fig MG-132 does not increase cell death in A549 cells. Cell death was determined via % viarypanblue inclusion in A549 cells with MG-132. Control cells were incubated with DMSO.n = 5, n.s. not significant.
(TIFF)
