# Correction: Detrimental ELAVL-1/HuR-dependent GSK3β mRNA stabilization impairs resolution in acute respiratory distress syndrome

**DOI:** 10.1371/journal.pone.0176134

**Published:** 2017-04-13

**Authors:** Olivia Hoffman, Nana Burns, István Vadász, Holger K. Eltzschig, Michael G. Edwards, Christine U. Vohwinkel

There is an error in reference 16. The correct reference is:

Sechler M, Borowicz S, Van Scoyk M, Avasarala S, Zerayesus S, Edwards MG, et al. Novel role for γ-catenin in the regulation of cancer cell migration via the induction of hepatocyte growth factor activator inhibitor type 1 (HAI-1). Journal of Biological Chemistry. 2015 Jun 19;290(25): 15610–20.
