# Supplementary material for: Detrimental ELAVL-1/HuR-dependent GSK3β mRNA stabilization impairs resolution in acute respiratory distress syndrome
Source: PLoS One. 2017 Feb 14;12(2):e0172116. doi: 10.1371/journal.pone.0172116 (PMC5308835; doi:10.1371/journal.pone.0172116)
Supplement: S1 Table — (PDF) [file pone.0172116.s001.pdf]

S1 Table

Probeset ID	Entrez Gene	Gene Symbol	Gene Title	RefSeq Transcrip	GEO# GSE3613 r-value	p-value
225886_at	1655	DDX5	DEAD (Asp-Glu-Ala-Asp)	NM_004396	0.714499	3.14E-27
229969_at	11231	SEC63	SEC63 homolog (S. cere	NM_007214 ///	0.68602	2.01E-24
227448_at	55082	ARGLU1	arginine and glutamate	NM_018011	0.663475	2.03E-22
226191_at	2932	GSK3B	glycogen synthase kinas	NM_001146156	0.749808	3.17E-31
227730_at	---	---	---	---	0.664286	1.73E-22
226018_at	222166	C7orf41	chromosome 7 open re	NM_152793	0.656487	7.83E-22
239300_at	5289	PIK3C3	phosphoinositide-3-kin	NM_002647	0.653535	1.37E-21
228310_at	55740	ENAH	enabled homolog (Dros	NM_001008493	0.688651	1.14E-24
226397_s_at	---	---	---	---	0.644775	6.96E-21
209068_at	9987	HNRPDL	heterogeneous nuclear	NM_001207000	0.661203	3.16E-22
201298_s_at	55233	MOB1A	MOB kinase activator 1	NM_018221	-0.669911	5.67E-23
224722_at	57534	MIB1	mindbomb E3 ubiquitin	NM_020774	0.648041	3.82E-21
227746_at	1994	ELAVL1	ELAV (embryonic lethal,	NM_001419	0.67614	1.60E-23
225111_s_at	63908	NAPB	N-ethylmaleimide-sensi	NM_022080	0.610329	2.56E-18
235725_at	4089	SMAD4	SMAD family member 4	NM_005359	0.626563	1.73E-19
238078_at	26984	SEC22A	SEC22 vesicle trafficking	NM_012430	0.598833	1.57E-17
223119_s_at	55031	USP47	ubiquitin specific peptic	NM_017944	0.630728	8.47E-20
228905_at	5108	PCM1	pericentriolar material 1	NM_006197	0.611625	2.08E-18
225017_at	64770	CCDC14	coiled-coil domain cont	NM_022757	0.606372	4.82E-18
200816_s_at	5048	PAFAH1B1	platelet-activating facto	NM_000430	0.608473	3.45E-18
201543_s_at	56681	SAR1A	SAR1 homolog A (S. cer	NM_001142648	-0.624742	2.36E-19
235224_s_at	55832	CAND1	cullin-associated and ne	NM_018448	0.646469	5.11E-21
235782_at	---	---	---	---	0.648333	3.62E-21
222918_at	51209	RAB9B	RAB9B, member RAS or	NM_016370	0.595016	2.83E-17
227728_at	5494	PPM1A	protein phosphatase, M	NM_021003 ///	0.624955	2.28E-19
202561_at	8658	TNKS	tankyrase, TRF1-interac	NM_003747	0.606455	4.76E-18
229705_at	5289	PIK3C3	phosphoinositide-3-kin	NM_002647	0.604982	6.01E-18
226186_at	29767	TMOD2	tropomodulin 2 (neuror	NM_001142885	0.608229	3.59E-18
244660_at	1994	ELAVL1	ELAV (embryonic lethal,	NM_001419	0.638704	2.08E-20
213852_at	9939	RBM8A	RNA binding motif prote	NM_005105	0.621086	4.38E-19
205933_at	26040	SETBP1	SET binding protein 1	NM_001130110	0.614283	1.35E-18
238620_at	---	---	---	---	0.604969	6.02E-18
228810_at	151195	CCNYL1	cyclin Y-like 1	NM_001142300	-0.613386	1.56E-18
232007_at	55326	AGPAT5	1-acylglycerol-3-phosph	NM_018361	0.615399	1.12E-18
219549_s_at	10313	RTN3	reticulon 3	NM_001265589	0.718166	1.29E-27
213411_at	53616	ADAM22	ADAM metallopeptidas	NM_004194 ///	0.644835	6.89E-21
244177_at	---	---	---	---	0.656457	7.88E-22
226713_at	152137	CCDC50	coiled-coil domain cont	NM_174908 ///	0.664278	1.73E-22
237299_at	---	---	---	---	0.595795	2.51E-17
228630_at	7637	ZNF84	zinc finger protein 84	NM_001127372	0.595807	2.50E-17
238992_at	11201	POLI	polymerase (DNA direct	NM_007195	0.628354	1.28E-19
243023_at	---	---	---	---	0.597518	1.93E-17
211929_at	220988	HNRNPA3	heterogeneous nuclear	NM_194247	0.627671	1.43E-19
242590_at	---	---	---	---	0.571077	9.45E-16
228967_at	10209	EIF1	eukaryotic translation ir	NM_005801	0.579851	2.70E-16
218184_at	56995	TULP4	tubby like protein 4	NM_001007466	0.57379	6.44E-16

218890_x_at		51318	MRPL35	mitochondrial ribosoma	NM_016622 ///	-0.613149	1.62E-18
235603_at		3192	HNRNPU	heterogeneous nuclear	NM_004501 ///	0.587004	9.45E-17
219662_at		79074	C2orf49	chromosome 2 open rea	NM_024093	-0.673128	2.96E-23
232019_at		342357	ZKSCAN2	zinc finger with KRAB ar	NM_001012981	0.581525	2.12E-16
227478_at		26040	SETBP1	SET binding protein 1	NM_001130110	0.56891	1.28E-15
225923_at		9217	VAPB	VAMP (vesicle-associate	NM_001195677	0.597424	1.95E-17
230904_at		83856	FSD1L	fibronectin type III and :	NM_001145313	0.585359	1.21E-16
233068_at	---		---	---	---	0.644431	7.41E-21
235611_at		140890	SREK1	splicing regulatory gluta	NM_001077199	0.598131	1.75E-17
212534_at		7572	ZNF24	zinc finger protein 24	NM_006965	0.646494	5.08E-21
227572_at		84749	USP30	ubiquitin specific peptic	NM_032663	0.57241	7.83E-16
208872_s_at		7905	REEP5	receptor accessory prot	NM_005669	-0.582637	1.80E-16
230178_s_at		55250	ELP2	elongation protein 2 ho	NM_001242875	0.62101	4.43E-19
225031_at		84181	CHD6	chromodomain helicase	NM_032221	0.573171	7.03E-16
226774_at		84498	FAM120B	family with sequence si	NM_032448	0.562233	3.22E-15
225704_at		57666	FBRSL1	fibrosin-like 1	NM_001142641	0.618542	6.69E-19
207625_s_at		9139	CBFA2T2	core-binding factor, run	NM_001032999	0.623134	3.10E-19
238115_at		202052	DNAJC18	DnaJ (Hsp40) homolog,	NM_152686	0.587035	9.40E-17
222646_s_at		30001	ERO1L	ERO1-like (S. cerevisiae)	NM_014584	-0.610648	2.43E-18
229651_at		124925	SEZ6	seizure related 6 homol	NM_001098635	0.580839	2.34E-16
230020_at		51691	NAA38	N(alpha)-acetyltransfera	NM_016200	0.61517	1.17E-18
226276_at		153339	TMEM167A	transmembrane proteir	NM_174909	-0.616228	9.80E-19
228548_at		5906	RAP1A	RAP1A, member of RAS	NM_001010935	0.625145	2.21E-19
227908_at		57465	TBC1D24	TBC1 domain family, me	NM_001199107	0.579083	3.02E-16
228788_at		29799	YPEL1	yippee-like 1 (Drosophil	NM_013313	0.622529	3.43E-19
204743_at		29114	TAGLN3	transgelin 3	NM_001008272	0.551343	1.39E-14
217856_at		9939	RBM8A	RNA binding motif prote	NM_005105	0.619938	5.30E-19
241114_s_at	---		---	---	---	0.624669	2.39E-19
230154_at		51322	WAC	WW domain containing	NM_016628 ///	0.629658	1.02E-19
222753_s_at		60559	SPCS3	signal peptidase comple	NM_021928	-0.554631	8.97E-15
230330_at		8493	PPM1D	protein phosphatase, M	NM_003620	0.596847	2.14E-17
238073_at		1996	ELAVL4	ELAV (embryonic lethal,	NM_001144774	0.558324	5.47E-15
226224_at		3607	FOXK2	forkhead box K2	NM_004514 ///	0.566541	1.78E-15
202172_at		7716	VEZF1	vascular endothelial zin	NM_007146	0.595584	2.59E-17
223523_at		66000	TMEM108	transmembrane proteir	NM_001136469	0.56366	2.65E-15
238043_at		57492	ARID1B	AT rich interactive doma	NM_017519 ///	0.57732	3.89E-16
223032_x_at		27166	PRELID1	PRELI domain containin	NM_013237	-0.562667	3.03E-15
212179_at		25957	PNISR	PNN-interacting serine/	NM_015491 ///	0.571475	8.93E-16
221727_at		10923	SUB1	SUB1 homolog (S. cerev	NM_006713	0.578132	3.46E-16
220999_s_at		26999	CYFIP2	cytoplasmic FMR1 inter	NM_001037332	0.596874	2.13E-17
205751_at		6456	SH3GL2	SH3-domain GRB2-like 2	NM_003026	0.562563	3.08E-15
227612_at		1995	ELAVL3	ELAV (embryonic lethal,	NM_001420 ///	0.56715	1.64E-15
227261_at		11278	KLF12	Kruppel-like factor 12	NM_007249 ///	0.552659	1.16E-14
202201_at		645	BLVRB	biliverdin reductase B (f	NM_000713	-0.570738	9.91E-16
224393_s_at		27439	CECR6	cat eye syndrome chror	NM_001163079	0.581038	2.27E-16
223362_s_at		55964	3-Sep	septin 3	NM_019106 ///	0.588876	7.15E-17
221207_s_at		26960	NBEA	neurobeachin	NM_001204197	0.560576	4.03E-15
206051_at		1996	ELAVL4	ELAV (embryonic lethal,	NM_001144774	0.555854	7.62E-15
217897_at		53826	FXYD6	FXYD domain containing	NM_001164831	0.582222	1.91E-16

211998_at	3020 /// 3021	H3F3A /// H3F	H3 histone, family 3A //	NM_002107 ///	0.608583	3.39E-18
231220_at	56853	CELF4	CUGBP, Elav-like family	NM_001025087	0.575882	4.78E-16
230706_s_at	94032	CAMK2N2	calcium/calmodulin-dep	NM_033259	0.607635	3.94E-18
229351_at	---	---	---	---	0.556839	6.68E-15
204995_at	8851	CDK5R1	cyclin-dependent kinase	NM_003885	0.57424	6.04E-16
230192_at	10206	TRIM13	tripartite motif containi	NM_001007278	0.570354	1.05E-15
213342_at	10413	YAP1	Yes-associated protein :	NM_001130145	-0.55834	5.46E-15
228502_at	27330	RPS6KA6	ribosomal protein S6 kir	NM_014496	0.597519	1.93E-17
206013_s_at	51412	ACTL6B	actin-like 6B	NM_016188	0.563514	2.70E-15
204662_at	9738	CCP110	centriolar coiled coil pro	NM_001199022	0.575113	5.33E-16
228792_at	---	---	---	---	0.589231	6.78E-17
225310_at	26787 /// 2731	RBMX /// SNO	RNA binding motif prote	NM_001164803	0.555242	8.27E-15
216033_s_at	2534	FYN	FYN oncogene related t	NM_001242779	0.572727	7.48E-16
222797_at	56896	DPYSL5	dihydropyrimidinase-lik	NM_001253723	0.582576	1.81E-16
203130_s_at	3800	KIF5C	kinesin family member :	NM_004522	0.562382	3.15E-15
233110_s_at	83596	BCL2L12	BCL2-like 12 (proline ric	NM_001040668	-0.558333	5.46E-15
227041_at	91404	SESTD1	SEC14 and spectrin dom	NM_178123	0.551961	1.28E-14
243624_at	9063	PIAS2	protein inhibitor of acti	NM_004671 ///	0.55944	4.70E-15
212498_at	---	---	---	---	0.560098	4.30E-15
213140_s_at	26039	SS18L1	synovial sarcoma transl	NM_015558 ///	0.579311	2.92E-16
223228_at	84247	LDOC1L	leucine zipper, down-re	NM_032287	0.567577	1.54E-15
208804_s_at	6431	SRSF6	serine/arginine-rich spli	NM_006275 ///	0.573255	6.94E-16
215143_at	349152	DPY19L2P2	dpy-19-like 2 pseudoge	NM_182634 ///	0.565262	2.12E-15
230821_at	7707	ZNF148	zinc finger protein 148	NM_021964	0.559366	4.75E-15
230238_at	134548	SOWAHA	sosondowah ankyrin re	NM_175873	0.55209	1.26E-14

3	GEO# GSE4824			
Rank	r-value	p-value	Rank	Ave Rank
3	0.785048	1.42E-15	1	2
5	0.724239	2.02E-12	2	3.5
11	0.694518	3.62E-11	3	7
1	0.656958	8.77E-10	13	7
9	0.657821	8.19E-10	11	10
13	0.662775	5.51E-10	8	10.5
15	0.668654	3.41E-10	7	11
4	0.643504	2.47E-09	18	11
21	0.684587	8.81E-11	5	13
12	0.652004	1.29E-09	14	13
8	-0.641503	2.87E-09	19	13.5
17	0.657186	8.61E-10	12	14.5
6	0.609672	2.70E-08	45	25.5
47	0.661975	5.88E-10	9	28
28	0.623551	1.05E-08	29	28.5
56	0.692077	4.52E-11	4	30
24	0.617993	1.54E-08	36	30
45	0.647923	1.77E-09	17	31
53	0.657904	8.14E-10	10	31.5
49	0.648882	1.64E-09	16	32.5
31	-0.619211	1.41E-08	34	32.5
19	0.607161	3.18E-08	51	35
16	0.603308	4.10E-08	55	35.5
66	0.671763	2.64E-10	6	36
30	0.611559	2.38E-08	42	36
52	0.637429	3.87E-09	21	36.5
54	0.636853	4.04E-09	22	38
50	0.626201	8.69E-09	27	38.5
23	0.60448	3.80E-08	54	38.5
35	0.611553	2.38E-08	43	39
42	0.617934	1.54E-08	37	39.5
55	0.627526	7.91E-09	26	40.5
43	-0.615659	1.80E-08	39	41
40	0.609085	2.80E-08	47	43.5
2	0.575528	2.30E-07	89	45.5
20	0.590197	9.44E-08	72	46
14	0.583626	1.41E-07	79	46.5
10	0.578627	1.91E-07	83	46.5
64	0.623451	1.05E-08	30	47
63	0.618546	1.48E-08	35	49
26	0.588616	1.04E-07	74	50
59	0.609782	2.68E-08	44	51.5
27	0.584098	1.37E-07	78	52.5
92	0.651753	1.32E-09	15	53.5
78	0.622746	1.11E-08	31	54.5
86	0.630081	6.60E-09	24	55

44	-0.597743	5.87E-08	66	55
70	0.611933	2.32E-08	41	55.5
7	-0.561412	5.21E-07	104	55.5
75	0.617705	1.57E-08	38	56.5
95	0.637538	3.84E-09	20	57.5
60	0.602976	4.19E-08	57	58.5
71	0.608906	2.84E-08	48	59.5
22	0.56632	3.94E-07	98	60
57	0.59974	5.16E-08	64	60.5
18	0.561724	5.12E-07	103	60.5
90	0.622221	1.15E-08	32	61
72	-0.607163	3.18E-08	50	61
36	0.576195	2.21E-07	87	61.5
88	0.613379	2.10E-08	40	64
105	0.62878	7.24E-09	25	65
38	0.56914	3.35E-07	95	66.5
33	0.564457	4.38E-07	102	67.5
69	0.596949	6.17E-08	67	68
46	-0.572248	2.79E-07	91	68.5
77	0.601238	4.69E-08	61	69
41	0.566263	3.95E-07	99	70
39	-0.565712	4.08E-07	101	70
29	0.554734	7.57E-07	112	70.5
80	0.601122	4.72E-08	62	71
34	0.557352	6.55E-07	108	71
120	0.634516	4.79E-09	23	71.5
37	0.559625	5.76E-07	106	71.5
32	0.554845	7.53E-07	111	71.5
25	0.551349	9.12E-07	118	71.5
116	-0.623644	1.04E-08	28	72
62	0.579012	1.87E-07	82	72
112	0.619662	1.37E-08	33	72.5
98	0.607416	3.13E-08	49	73.5
65	0.57739	2.06E-07	86	75.5
100	0.606284	3.37E-08	52	76
82	0.58964	9.78E-08	73	77.5
102	-0.602753	4.25E-08	58	80
91	0.593443	7.71E-08	69	80
81	0.582491	1.52E-07	80	80.5
61	0.565911	4.03E-07	100	80.5
103	0.602331	4.37E-08	59	81
97	0.598888	5.45E-08	65	81
117	0.609464	2.73E-08	46	81.5
93	-0.590479	9.28E-08	71	82
76	0.575738	2.27E-07	88	82
68	0.56868	3.44E-07	96	82
106	0.602148	4.42E-08	60	83
114	0.605215	3.62E-08	53	83.5
74	0.570539	3.09E-07	93	83.5

48	0.550778	9.41E-07	119	83.5
83	0.577434	2.06E-07	85	84
51	0.551757	8.92E-07	117	84
113	0.603212	4.12E-08	56	84.5
85	0.578065	1.98E-07	84	84.5
94	0.587003	1.15E-07	76	85
110	-0.600689	4.86E-08	63	86.5
58	0.5521	8.76E-07	116	87
101	0.587927	1.09E-07	75	88
84	0.566811	3.83E-07	97	90.5
67	0.553336	8.18E-07	114	90.5
115	0.595029	6.97E-08	68	91.5
89	0.570435	3.10E-07	94	91.5
73	0.556562	6.84E-07	110	91.5
104	0.58036	1.72E-07	81	92.5
111	-0.585607	1.25E-07	77	94
119	0.590854	9.06E-08	70	94.5
108	0.574006	2.52E-07	90	99
107	0.570641	3.07E-07	92	99.5
79	0.550689	9.46E-07	120	99.5
96	0.560413	5.51E-07	105	100.5
87	0.553282	8.20E-07	115	101
99	0.558931	5.99E-07	107	103
109	0.556566	6.84E-07	109	109
118	0.553643	8.04E-07	113	115.5
