# Supplementary figures and images for: Detrimental ELAVL-1/HuR-dependent GSK3β mRNA stabilization impairs resolution in acute respiratory distress syndrome
Source: PLoS One. 2017 Feb 14;12(2):e0172116. doi: 10.1371/journal.pone.0172116 (PMC5308835; doi:10.1371/journal.pone.0172116)

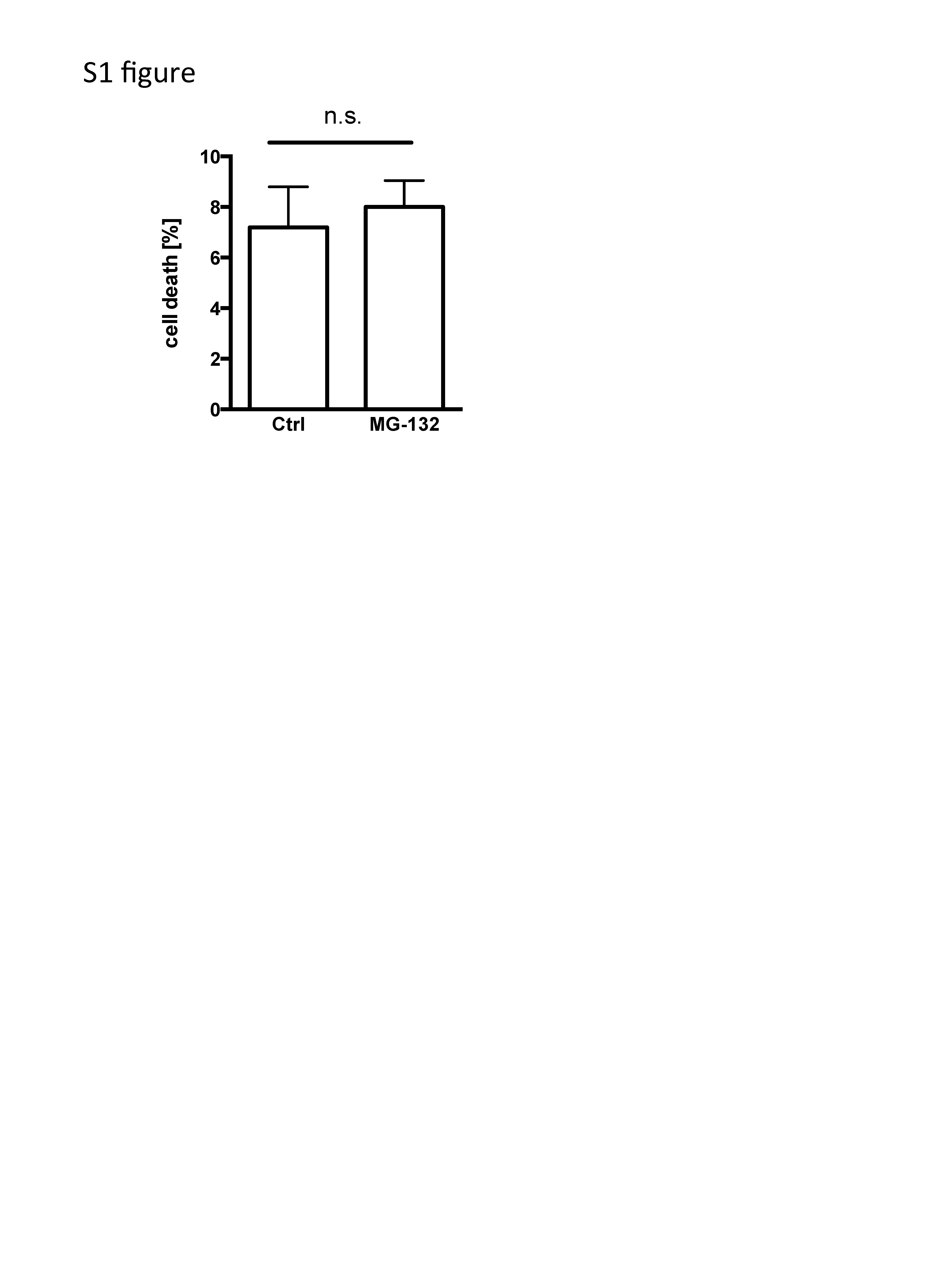

Supplement: S1 Fig — Cell death was determined via % viarypanblue inclusion in A549 cells with MG-132. Control cells were incubated with DMSO.n = 5, n.s. not significant. (TIFF) [file pone.0172116.s002.tiff]
